# Adverse events associated with amlodipine: a pharmacovigilance study using the FDA adverse event reporting system

**DOI:** 10.3389/fcvm.2025.1504671

**Published:** 2025-05-06

**Authors:** Jiazhen Jiang, Qian Zhong, Xinyu Zhou, Lisi Zhou, Jiyuan Zheng, Bingshuo Liu, Xingwei Di

**Affiliations:** ^1^ The First Clinical Medical School of Guangzhou University of Chinese Medicine, Guangzhou, Guangdong, China; ^2^ Department of Cardiovascular Medicine, First Affiliated Hospital of Jinzhou Medical University, Jinzhou, Liaoning, China; ^3^ Clinical Medical College of Acupuncture, Moxibustion and Rehabilitation, Guangzhou University of Chinese Medicine, Guangzhou, Guangdong, China; ^4^ The Fifth Clinical College, Guangzhou University of Chinese Medicine, Guangzhou, Guangdong, China; ^5^ Department of Cardiovascular Medicine, The First Affiliated Hospital of Guangzhou University of Chinese Medicine, Guangzhou, Guangdong, China

**Keywords:** amlodipine, adverse drug reactions, FDA adverse event reporting system, risk signal detection, logistic regression

## Abstract

**Background:**

Amlodipine, a widely prescribed clinical medication, is associated with adverse reactions that can impede the proper execution of treatment regimens. The lack of systematic studies on amlodipine's adverse drug reactions (ADRs) necessitates further investigation to facilitate refined population management and optimize therapeutic outcomes.

**Method:**

This study leveraged the FDA Adverse Event Reporting System (FAERS) database, extracting reports submitted exclusively by healthcare professionals where amlodipine was designated as the primary suspect (PS). Four risk signal detection methods were employed: Ratio of Odds Ratio, Proportional Reporting Ratio, Bayesian Confidence Propagation Neural Network, and Empirical Bayes Geometric Mean, to conduct a comprehensive analysis of amlodipine-related ADRs. Furthermore, subgroup analyses stratified by gender and age were performed, with multivariable logistic regression utilized to validate the reliability of the findings.

**Results:**

Across the general population, male cohort, female cohort, elderly group, and younger demographic, the four signal detection methods collectively identified 513, 348, 403, 246, and 260 potential ADRs associated with amlodipine, respectively. Intersection analysis revealed 27 common ADRs, including gingival hypertrophy, vasoplegia syndrome, and distributive shock. Subsequent multivariable logistic regression confirmed amlodipine's role as an independent risk factor for all 27 ADRs (OR > 1, *P* < 0.05).

**Conclusion:**

This study provides compelling evidence that amlodipine poses risks of peripheral edema, shock, and dyspnea, among others. Additionally, it identified previously unreported ADRs such as abnormal full blood count and personality disorder. These findings underscore the importance of exercising caution when prescribing amlodipine to high-risk individuals with a history of hyperkalemia, cardiac structural abnormalities, or airway obstruction.

## 1 Introduction

Amlodipine, a widely prescribed dihydropyridine calcium channel blocker, has been predominantly utilized in the treatment of hypertension, coronary artery disease, and other cardiovascular ailments since its approval in 1991 (1). As a calcium channel antagonist, amlodipine exerts its therapeutic effect by inhibiting calcium ion influx into vascular smooth muscle cells, thereby inducing vasodilation and reducing peripheral vascular resistance, ultimately leading to a decrease in blood pressure (2). Among antihypertensive medications, calcium channel blockers (CCBs) are the most frequently prescribed, with amlodipine accounting for a substantial 37% of prescriptions (3), underscoring its extensive clinical application. Amlodipine's pharmacokinetic profile, characterized by low renal clearance (7 ml/min/mg), extended half-life (35–50 h), and high bioavailability (60%–80%) (4), renders it suitable for once-daily dosing, a feature highly favored by clinicians.

Notwithstanding its widespread use, an observational study revealed that amlodipine, when used as monotherapy, is associated with the highest incidence of adverse reactions among antihypertensive agents (5). The FDA label indicates that the most common adverse effects of amlodipine include edema, dizziness, flushing, and palpitations. Recent years have witnessed the emergence of additional adverse reactions, including acute kidney injury (6), thrombocytopenia (7), bradycardia (1), dermatological complications (8–10), gingival hyperplasia (11), and even shock (12). These adverse effects not only directly impact patients' quality of life and increase the likelihood of treatment discontinuation but may also pose life-threatening risks in severe cases (13), presenting significant challenges to the clinical application of amlodipine.

While reports of amlodipine-related adverse reactions are on the rise, the majority of these accounts stem from individual case reports, lacking robust supporting evidence. Moreover, accurate assessment of drug-specific adverse reactions in clinical observations is hampered by limited subject numbers and short observation periods. Consequently, signal generation based on large-scale databases has emerged as a crucial method for detecting adverse drug reactions (14). This study represents the first systematic analysis of amlodipine-related adverse reactions grounded in real-world data and employing multiple methodological approaches. The adverse reactions identified have been corroborated through four distinct methods to enhance credibility. The primary objective is to assist clinicians in recognizing potential clinical adverse reactions, thereby strengthening the monitoring and refined management of amlodipine therapy, which is paramount for optimizing treatment outcomes.

## 2 Materials and methods

### 2.1 Data source

This observational analysis utilizes the FDA Adverse Event Reporting System (FAERS) database, which is updated quarterly and comprises self-reported data from both healthcare professionals (physicians, pharmacists, healthcare specialists, and registered nurses) and non-healthcare professionals (consumers, lawyers, sales representatives, and others). The FAERS database has been extensively employed in identifying potential drug adverse reactions. It encompasses unique identification numbers, report dates, reporting countries, primary reporter qualifications, patient demographic information (such as gender, age, and weight), suspected and concomitant medications and their indications, ADR occurrence dates, and ADR manifestations.

Given amlodipine's market approval in 1991, this study extracted report files from the FAERS database (https://fis.fda.gov/extensions/FPD-QDE-FAERS/FPD-QDE-FAERS.html) spanning from the database's inception (Q1 2004) to Q2 2024. Rigorous deduplication was performed, particularly focusing on eliminating overlapping information in key fields such as AE, event date, gender, age, weight, reporting country, and primary suspected active substance. To mitigate false positives arising from potential misreporting due to lack of professional knowledge, this study exclusively included reports submitted by healthcare professionals. To address issues of duplicate reporting and non-standardized drug nomenclature, the research team compiled a comprehensive list of amlodipine's drug and brand names, meticulously reorganizing drug name variants within the database. Drug entries were strictly limited to amlodipine, excluding other medications such as nimodipine. The curation process was independently conducted by two researchers, with discrepancies resolved by a third researcher. Only reports involving amlodipine as the primary suspected agent were retained for analysis.

### 2.2 Disproportionality analysis

**Figure 1:**
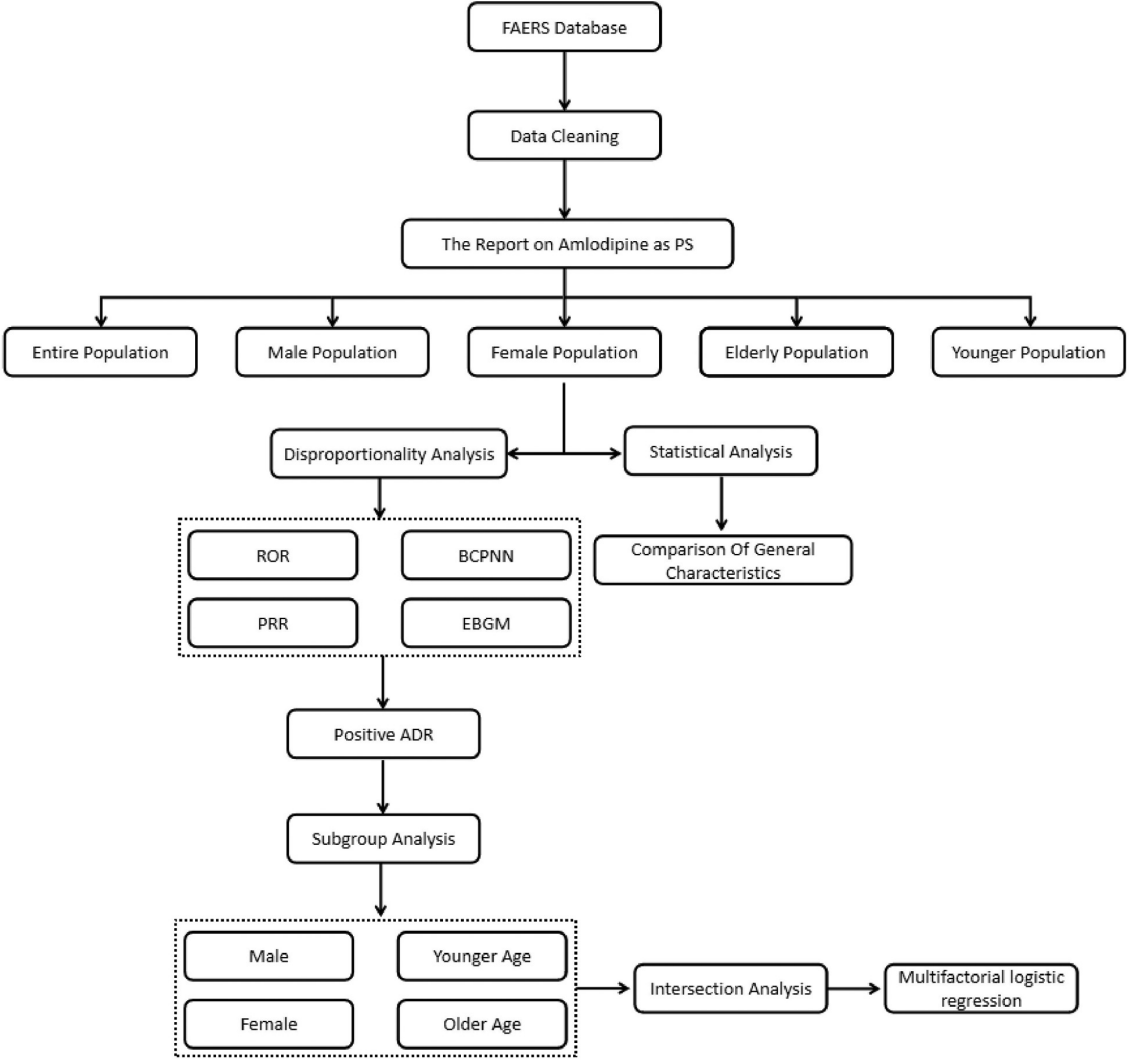
Flowchart of the study design and analysis pipeline.

To robustly ascertain the ADRs associated with amlodipine, this study employed a signal disproportionate analysis framework, integrating four risk signal detection methodologies: Ratio of Odds Ratio (ROR) (15), Proportional Reporting Ratio (PRR) (16), Bayesian Confidence Propagation Neural Network (BCPNN) (17), and Empirical Bayes Geometric Mean (EGBM) (18). The detection criteria are delineated in Table 1. For this study, an ADR was deemed a potential adverse reaction to amlodipine only if all four algorithms identified a signal for that specific ADR. Furthermore, the analysis was stratified by gender and age, facilitating subgroup analyses that subsequently identified both common and unique ADRs within different populations, thereby providing evidence for the refined monitoring of various clinical cohorts. The flowchart illustrating this process can be found in Figure 1.

**Table 1:** Formulas and criteria for identifying safety signals in four methods.

Category	Formula/Criteria	Target drug	Not-target drug
Target ADR		a	c
Not-target ADR		b	d
Total	*N* = a + b + c + d		
ROR PRR BCPNN EGBM	ROR = (ad)/(bc)	PRR = [a(c + d)]/[c(a + b)]	
	95% CI = eln(ROR) + 1.96√(1/a + 1/b + 1/c + 1/d)	X² = N(ad-bc)2/[(a + b)(c + d)(a + c)(b + d)	
	The criteria of positive safety signal detection by ROR: the lower limit of 95% CI >1, a ≥3	The criteria of positive safety signal detection by PRR: PRR ≥2, X² ≥4, N ≥ 3	
	IC = log2(aN)/(a + b)(a + c)	EBGM = (aN)/[a + b)(a + c)]	
	95% CI = E(IC) ± 2*√V(IC)	95% CI = eln(EGBM) ± 1.96√(1/a + 1/b + 1/c + 1/d)	
	The criteria of positive safety signal detection of BPCNN:IC025 > 0 (IC025: the lower bound of 95% CD)	The positive safety signal detection criteria by EGBM:EBGM05 > 2 (EBGM05: the lower bound of 95% CI)	

## 3 Results

### 3.1 Basic characteristics

Between the first quarter of 2004 and the second quarter of 2024, we retrieved 18,886 reports from the FAERS database, wherein amlodipine was designated as the primary suspect (PS). Among these reports, a majority were from female patients (48.6%), with individuals aged 65 and older constituting the predominant demographic (42.1%). This suggests that the use of amlodipine in the elderly population warrants significant attention. The top five reporting countries were the United States, United Kingdom, Canada, France, and Italy, with detailed results presented in Table 2.

**Table 2:** General characteristics table.

Variable	Amlodipine(*N* = 18,886)
Gender	
Female	9,182 (48.6%)
Male	7,846 (41.5%)
Unknown	1,858 (9.8%)
Weight	
<50 kg	398 (2.1%)
>100 kg	702 (3.7%)
50–100 kg	4,143 (21.9%)
Unknown	13,643 (72.2%)
Age	
<18	695 (3.7%)
≥86	1,217 (6.4%)
18–64	7,170 (38.0%)
65–85	6,737 (35.7%)
Unknown	3,067 (16.2%)
Reporter	
Health Professional	5,291 (28.0%)
Medical Doctor	10,038 (53.2%)
Pharmacist	3,549 (18.8%)
Registered Nurse	8 (0.0%)
Top 5 Reporting Countries	
	United States
	United Kingdom
	Canada
	France
	Italy

### 3.2 Identification of signal for ADRs in the entire population

**Figure 2:**
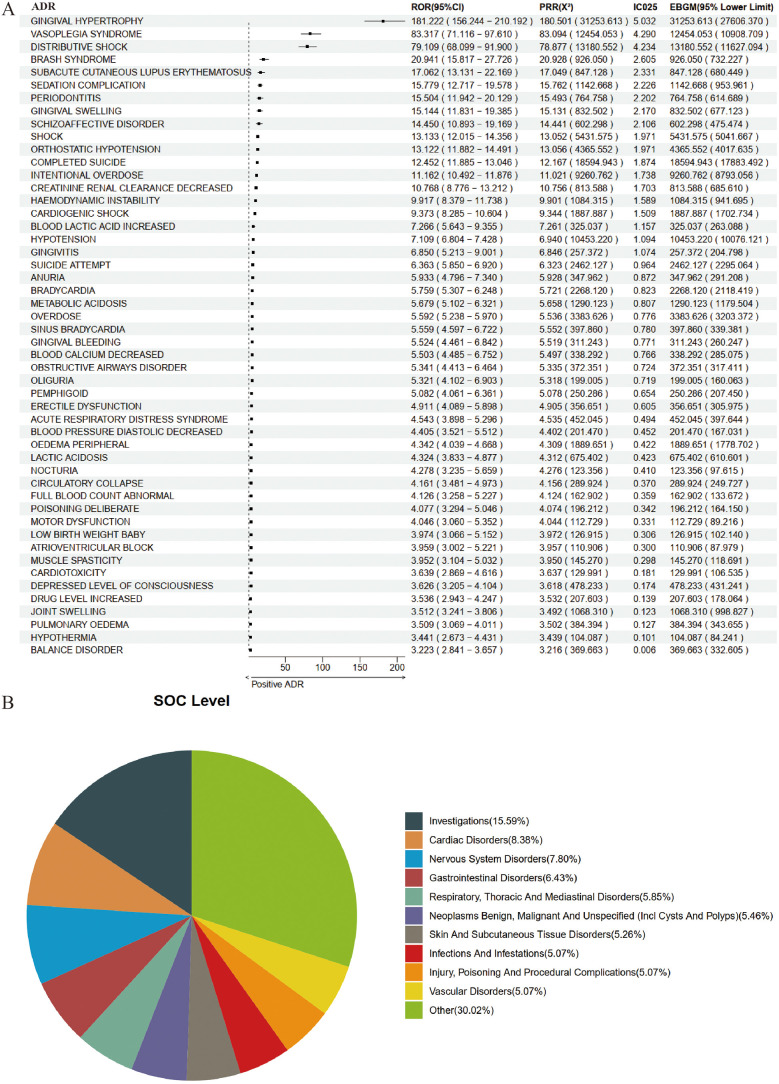
Positive signal detection in the entire epileptic population. **(A)** Forest plot of top 50 positive adverse drug reactions (ADRs). **(B)** System organ class (SOC) mapping chart.

Based on 18,886 reports from the entire population, we utilized a combination of four methodologies to identify ADR signals associated with amlodipine, resulting in a total of 513 potential ADRs. The top five, ranked by ROR, were as follows: Hypoinsulinaemia [a: 7, ROR (95% CI lower limit): 85.321, PRR (X^2^): 243.233 (844.330), IC025: 5.107, EBGM (95% CI lower limit): 351.393]; Gingival Hypoplasia [a: 4, ROR (95% CI lower limit): 52.253, PRR (X^2^): 194.586 (427.981), IC025: 4.863, EBGM (95% CI lower limit): 142.440]; Gingival Hypertrophy [a: 305, ROR (95% CI lower limit): 156.244, PRR (X^2^): 180.501 (31253.613), IC025: 5.032, EBGM (95% CI lower limit): 27,606.370]; Subepidermal Haemorrhage [a: 5, ROR (95% CI lower limit): 41.57, PRR (X^2^): 121.616 (398.749), IC025: 4.532, EBGM (95% CI lower limit): 162.398]; and Increased Body Fluid [a: 4, ROR (95% CI lower limit): 36.622, PRR (X^2^): 121.616 (318.999), IC025: 4.499, EBGM (95% CI lower limit): 116.849]. The results for the top 50 are illustrated in Figure 2A.

When mapped to the System Organ Class (SOC) level, the potential ADRs associated with amlodipine in the general population predominantly encompassed Investigations (15.59%), Cardiac Disorders (8.38%), Nervous System Disorders (7.80%), Gastrointestinal Disorders (6.43%), and Respiratory, Thoracic and Mediastinal Disorders (5.85%) (Figure 2B).

### 3.3 Identification of signal for ADRs in male population

**Figure 3:**
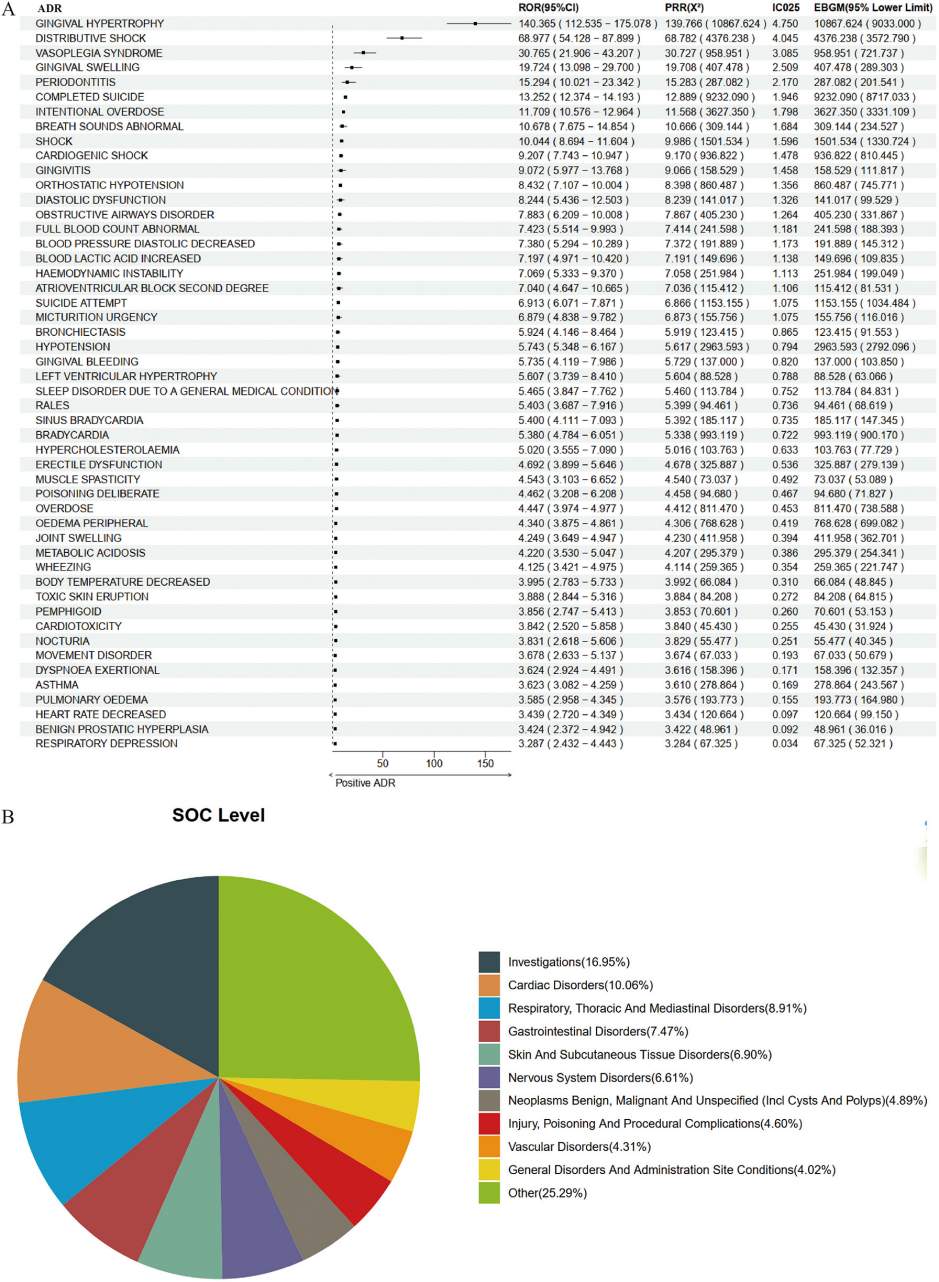
Positive signal detection in male epileptic population. **(A)** Forest plot of top 50 positive ADRs. **(B)** SOC mapping chart.

Based on 7,846 reports from the male population, we employed a combination of four methodologies to identify ADR signals associated with amlodipine, resulting in a total of 348 potential ADRs. The top five, ranked by ROR, were as follows: Electrocardiogram J Wave [a: 6, ROR (95% CI lower limit): 93.112, PRR (X^2^): 329.913 (787.000), IC025: 5.171, EBGM (95% CI lower limit): 273.027]; Increased Body Fluid [a: 4, ROR (95% CI lower limit): 47.252, PRR (X^2^): 175.954 (386.577), IC025: 4.718, EBGM (95% CI lower limit): 128.656]; Gingival Hypoplasia [a: 3, ROR (95% CI lower limit): 36.92, PRR (X^2^): 164.957 (279.365), IC025: 4.612, EBGM (95% CI lower limit): 79.831]; Cockroach Allergy [a: 3, ROR (95% CI lower limit): 36.92, PRR (X^2^): 164.957 (279.365), IC025: 4.612, EBGM (95% CI lower limit): 79.831]; and Gingival Hypertrophy [a: 129, ROR (95% CI lower limit): 112.535, PRR (X^2^): 139.766 (10,867.624), IC025: 4.750, EBGM (95% CI lower limit): 9,033.000]. The results for the top 50 are illustrated in Figure 3A.

When mapped to the SOC level, the potential ADRs associated with amlodipine in the male population predominantly encompassed Investigations (16.95%), Cardiac Disorders (10.06%), Respiratory, Thoracic and Mediastinal Disorders (8.91%), Gastrointestinal Disorders (7.47%), and Skin and Subcutaneous Tissue Disorders (6.90%) (Figure 3B).

### 3.4 Identification of signal for ADRs in female population

**Figure 4:**
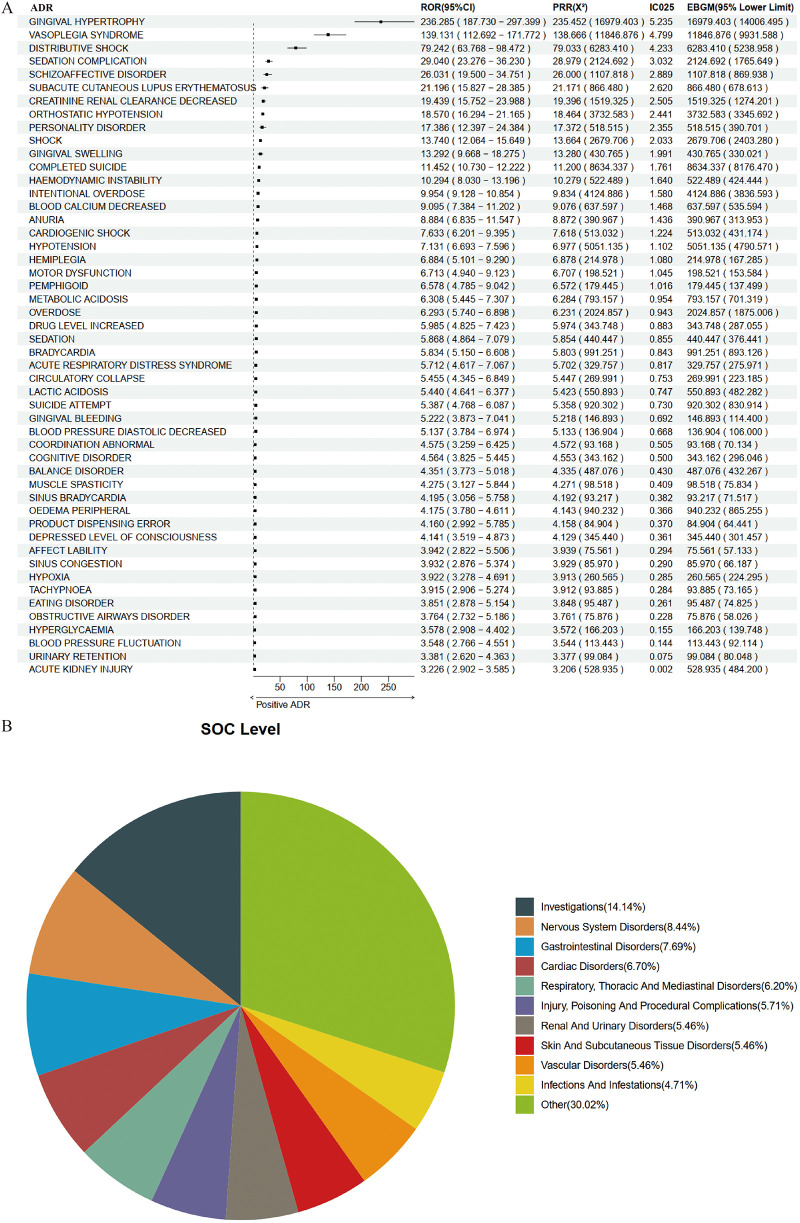
Positive signal detection in female epileptic population. **(A)** Forest plot of top 50 positive ADRs. **(B)** SOC mapping chart.

Based on 9,182 reports from the female population, we employed a combination of four methodologies to identify ADR signals associated with amlodipine, resulting in a total of 403 potential ADRs. The top five, ranked by ROR, were as follows: Friedreich's Ataxia [a: 4, ROR (95% CI lower limit): 72.722, PRR (X^2^): 324.912 (553.570), IC025: 5.174, EBGM (95% CI lower limit): 158.189]; Gingival Hypertrophy [a: 143, ROR (95% CI lower limit): 187.73, PRR (X^2^): 235.452 (16,979.403), IC025: 5.235, EBGM (95% CI lower limit): 14,006.495]; Malpositioned Teeth [a: 7, ROR (95% CI lower limit): 77.329, PRR (X^2^): 213.224 (788.587), IC025: 5.020, EBGM (95% CI lower limit): 337.452]; Congenital Acrochordon [a: 5, ROR (95% CI lower limit): 61.979, PRR (X^2^): 203.070 (548.387), IC025: 4.935, EBGM (95% CI lower limit): 203.137]; and Rectourethral Fistula [a: 5, ROR (95% CI lower limit): 61.979, PRR (X^2^): 203.070 (548.387), IC025: 4.935, EBGM (95% CI lower limit): 203.137]. The results for the top 50 are illustrated in Figure 4A.

When mapped to the SOC level, the potential ADRs associated with amlodipine in the female population predominantly encompassed Investigations (14.14%), Nervous System Disorders (8.44%), Gastrointestinal Disorders (7.69%), Cardiac Disorders (6.70%), and Respiratory, Thoracic and Mediastinal Disorders (6.20%) (Figure 4B).

### 3.5 Identification of signal for ADRs in elderly population

**Figure 5:**
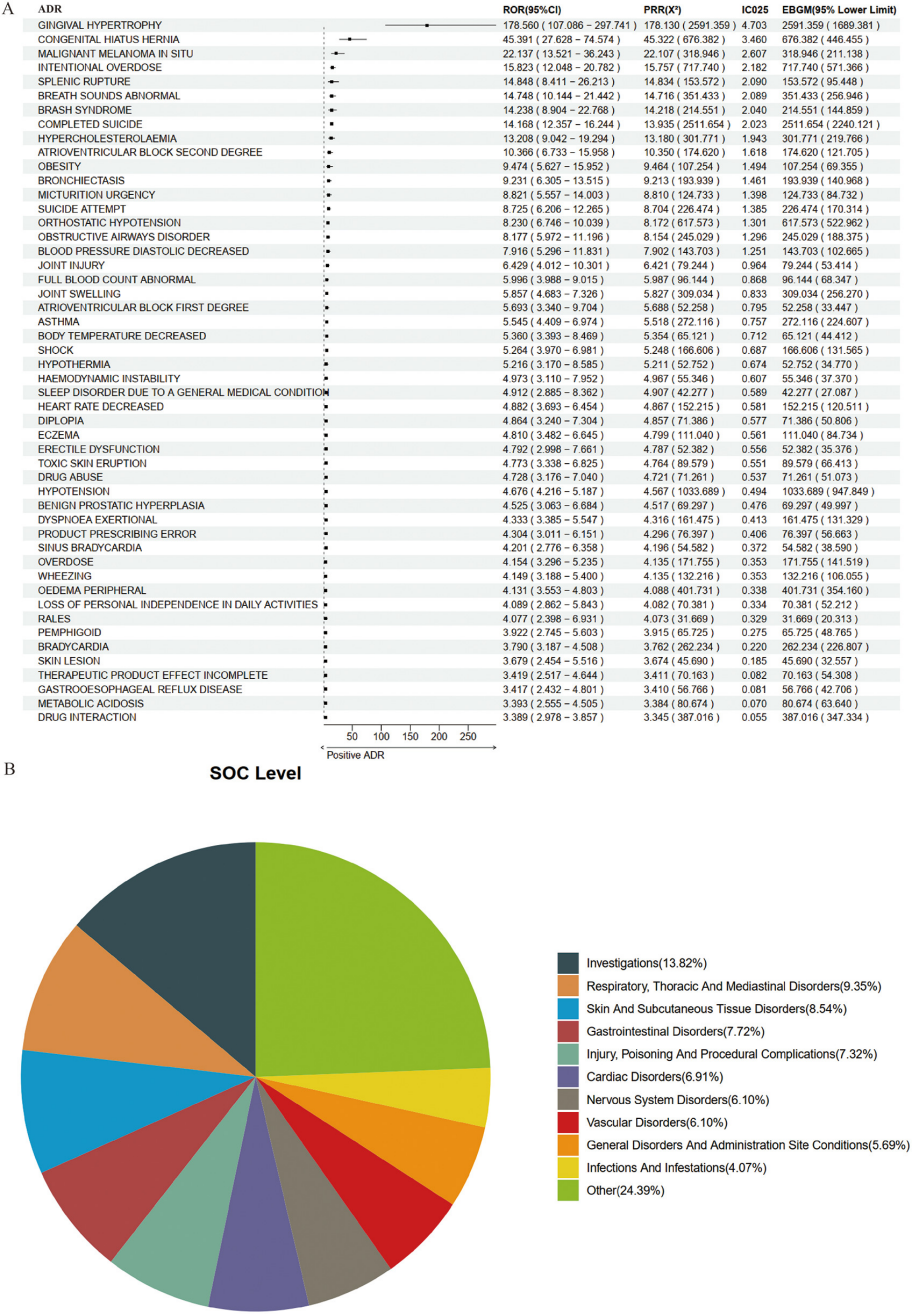
Positive signal detection in elderly epileptic population. **(A)** Forest plot of positive ADRs. **(B)** SOC mapping chart.

Based on 7,954 reports from the elderly population, we employed a combination of four methodologies to identify ADR signals associated with amlodipine, resulting in a total of 246 potential ADRs. The top five, ranked by ROR, were as follows: Cockroach Allergy [a: 3, ROR (95% CI lower limit): 50.214, PRR (X^2^): 482.675 (360.509), IC025: 4.817, EBGM (95% CI lower limit): 54.258]; Increased Body Fluid [a: 3, ROR (95% CI lower limit): 40.331, PRR (X^2^): 241.338 (287.211), IC025: 4.561, EBGM (95% CI lower limit): 64.266]; Gingival Hypertrophy [a: 31, ROR (95% CI lower limit): 107.086, PRR (X^2^): 178.130 (2,591.359), IC025: 4.703, EBGM (95% CI lower limit): 1,689.381]; Burning Feet Syndrome [a: 3, ROR (95% CI lower limit): 32.477, PRR (X^2^): 160.892 (238.348), IC025: 4.348, EBGM (95% CI lower limit): 62.464]; and Acute Biphenotypic Leukaemia [a: 5, ROR (95% CI lower limit): 40.929, PRR (X^2^): 134.076 (360.231), IC025: 4.339, EBGM (95% CI lower limit): 133.429]. The results for the top 50 are illustrated in Figure 5A.

When mapped to the SOC level, the potential ADRs associated with amlodipine in the elderly population predominantly encompassed Investigations (13.82%), Respiratory, Thoracic and Mediastinal Disorders (9.35%), Skin and Subcutaneous Tissue Disorders (8.54%), Gastrointestinal Disorders (7.72%), and Injury, Poisoning and Procedural Complications (7.32%) (Figure 5B).

### 3.6 Identification of signal for ADRs in younger population

**Figure 6:**
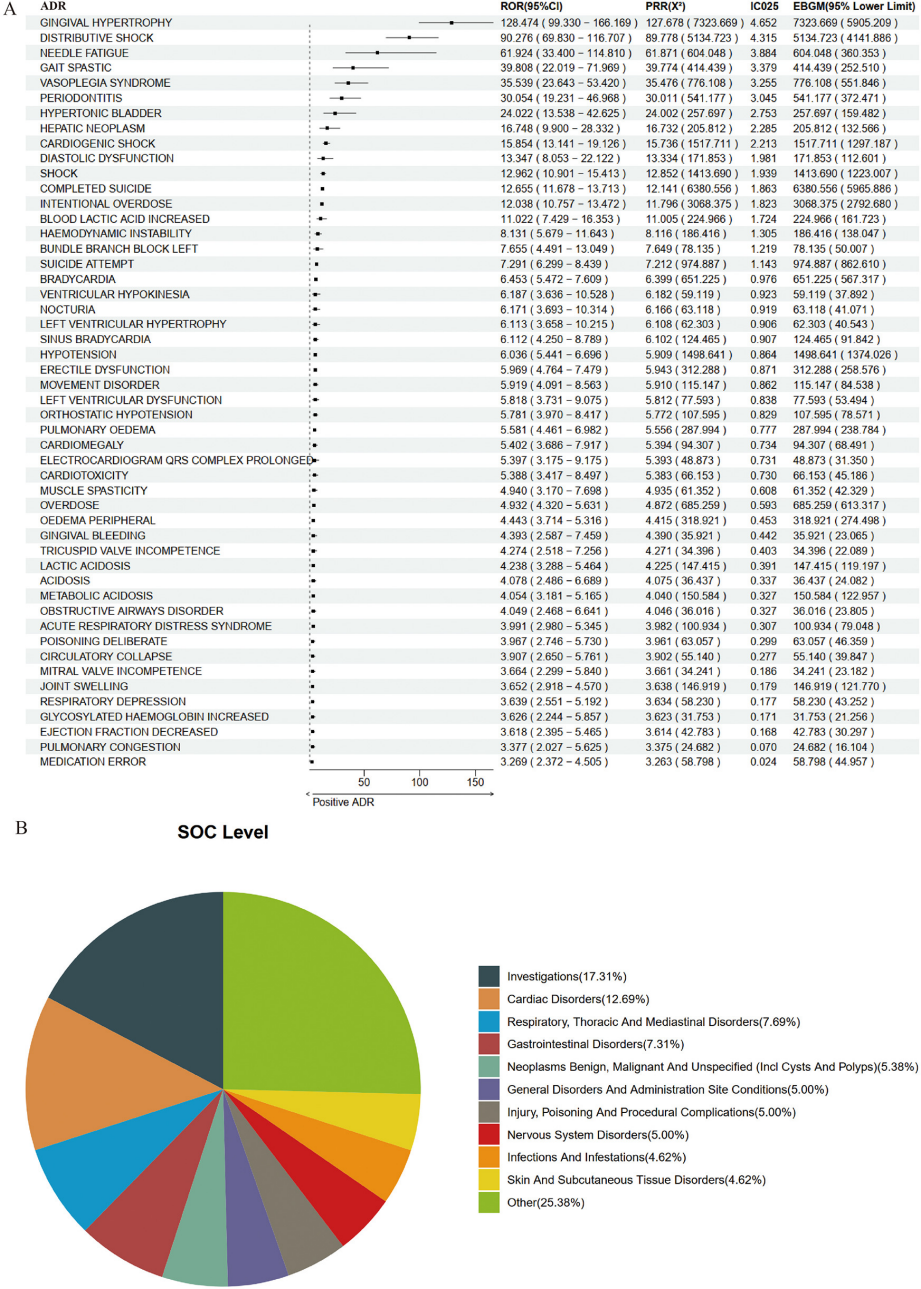
Positive signal detection in younger epileptic population. **(A)** Forest plot of top 50 positive ADRs. **(B)** SOC mapping chart.

Based on 7,865 reports from the younger population, we employed a combination of four methodologies to identify ADR signals associated with amlodipine, resulting in a total of 260 potential ADRs. The top five ranked by ROR were as follows: Electrocardiogram J Wave [a: 6, ROR (95% CI lower limit): 90.682, PRR (X^2^): 321.255 (766.221), IC025: 5.133, EBGM (95% CI lower limit): 265.807]; Hypoinsulinaemia [a: 3, ROR (95% CI lower limit): 53.685, PRR (X^2^): 321.255 (383.110), IC025: 4.972, EBGM (95% CI lower limit): 85.726]; Adrenal Cyst [a: 3, ROR (95% CI lower limit): 53.685, PRR (X^2^): 321.255 (383.110), IC025: 4.972, EBGM (95% CI lower limit): 85.726]; Gingival Hypoplasia [a: 3, ROR (95% CI lower limit): 43.231, PRR (X^2^): 214.170 (318.262), IC025: 4.758, EBGM (95% CI lower limit): 83.409]; and Oropharyngeal Oedema [a: 5, ROR (95% CI lower limit): 54.48, PRR (X^2^): 178.475 (481.312), IC025: 4.749, EBGM (95% CI lower limit): 178.280]. The results for the top 50 are illustrated in Figure 6A.

When mapped to the SOC level, the potential ADRs associated with amlodipine in the younger population predominantly encompassed Investigations (17.31%), Cardiac Disorders (12.69%), Respiratory, Thoracic and Mediastinal Disorders (7.69%), Gastrointestinal Disorders (7.31%), and Neoplasms—Benign, Malignant, and Unspecified (including Cysts and Polyps) (5.38%) (Figure 6B).

## 4 Intersection analysis

**Figure 7:**
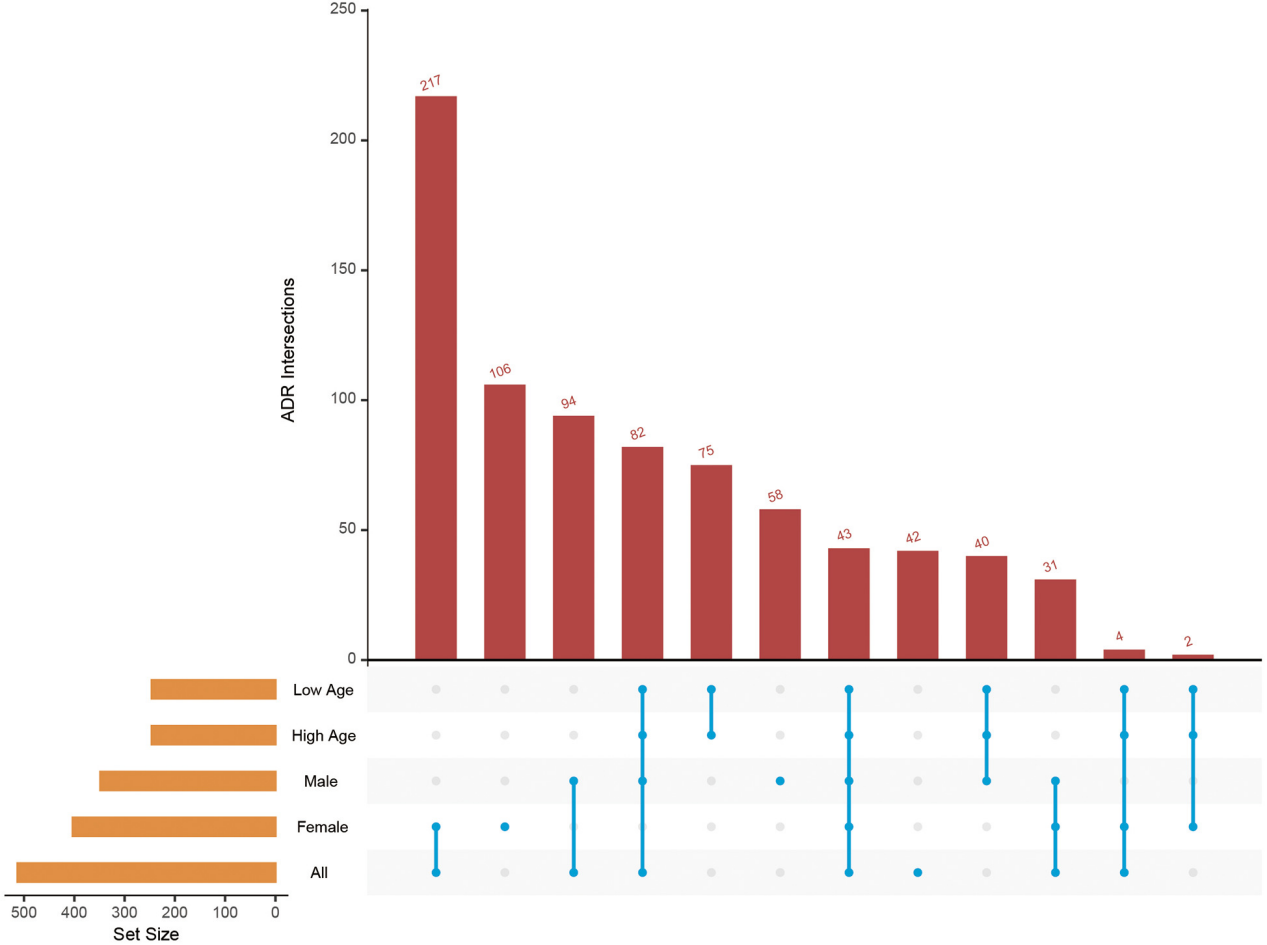
Upset plot of intersection ADRs across subgroups.

Through intersection analysis across various subgroup populations (the entire population, male population, female population, elderly population, and younger population), we identified 27 common ADRs associated with amlodipine: gingival hypertrophy, vasoplegia syndrome, distributive shock, myocardial depression, hyperdynamic left ventricle, sinus rhythm, gingival swelling, hyperplasia, shock, orthostatic hypotension, completed suicide, intentional overdose, hemodynamic instability, hypotension, gingivitis, suicide attempt, bradycardia, dyspnea at rest, metabolic acidosis, overdose, left ventricular hypertrophy, sinus bradycardia, gingival bleeding, obstructive airway disorder, gingival pain, decreased diastolic blood pressure, and peripheral edema. The primary focus of these ADRs is within the domains of cardiac disorders, respiratory issues, and dental and gingival conditions (Figure 7).

## 5 Multivariable logistic regression

**Figure 8:**
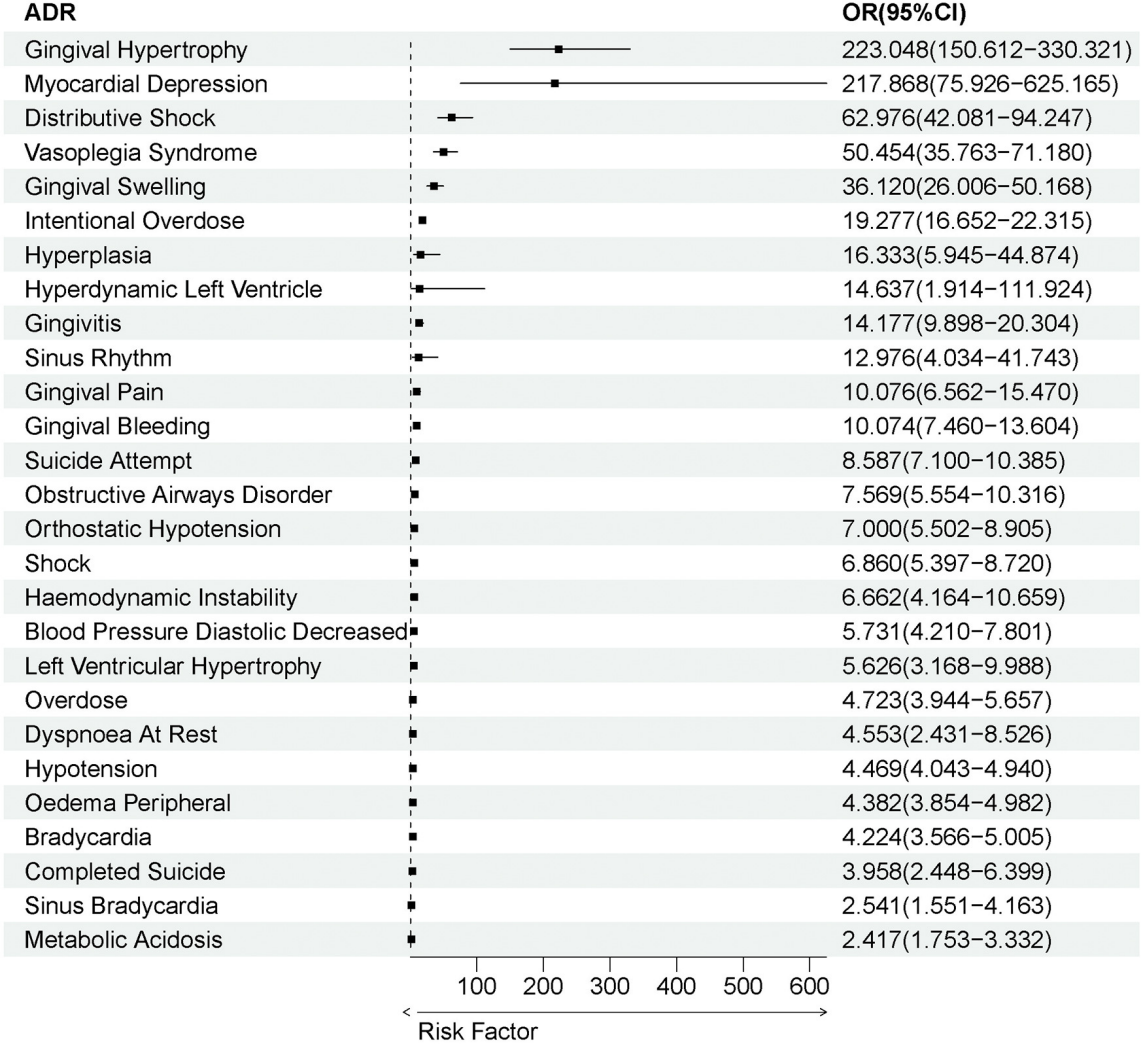
ADRs validated by multivariate logistic regression analysis.

To further ascertain whether the potential ADRs associated with amlodipine exert independent effects, we subsequently employed multivariable logistic regression, treating each of the 27 potential ADRs as binary outcome variables. The results indicate that amlodipine serves as an independent risk factor for the occurrence of all 27 potential ADRs (OR > 1), with statistically significant findings (*P* < 0.05). This suggests that these associations are not influenced by age or gender, aligning with the previously reported results (Figure 8).

## 6 Discussion

Amlodipine, a calcium channel blocker widely used in the treatment of hypertension and coronary artery disease, has demonstrated superior efficacy in controlling blood pressure variability compared to other calcium channel blockers. It has also shown cost-effectiveness relative to conventional therapies, leading to its recommendation as a preferred medication for patients with coronary artery disease (CAD) (19). Despite being recommended as a first-line therapy for hypertension, its use is constrained by potential side effects, and it has been reported as a primary cause of cardiovascular drug-related overdose deaths in the United States (20). Consequently, post-marketing surveillance of medications is crucial, particularly for commonly prescribed drugs like amlodipine.

This study represents the first comprehensive investigation of potential ADRs associated with amlodipine using the FAERS database. Employing disproportionality analysis, a rapid and economical method, we identified 27 common potential risks across various subgroups (including the general population, males, females, elderly, and younger individuals), which appear to be independent of age and gender. These findings were further validated through multivariable logistic regression, emphasizing their independence. This underscores the importance of vigilant monitoring for these potential ADRs in clinical applications of amlodipine, providing crucial evidence for clinical decision-making.

Peripheral edema, characterized by fluid retention in the extremities or other body parts, is a widely recognized and common adverse effect of amlodipine. It is generally attributed to the dilation of precapillary arterioles in the lower limbs, where increased hydrostatic pressure promotes fluid transfer into interstitial spaces. Amlodipine is used both as monotherapy and in combination with other antihypertensive medications. As monotherapy, statistical analyses indicate that amlodipine induces peripheral edema in 16.6% of cases (21), a higher incidence compared to other antihypertensive drugs (22). A specific clinical trial also found higher rates of peripheral and pulmonary edema in patients receiving amlodipine treatment compared to the control group (23). However, in the treatment of gestational hypertension, Yin et al. (24) found that amlodipine demonstrated superior efficacy to nifedipine, with reduced maternal side effect risks. Combination therapy with amlodipine has shown improved outcomes, such as significant blood pressure reduction and decreased incidence of peripheral edema when used with ACE inhibitors or diuretics (22, 25). Its combination with aliskiren exhibited enhanced antihypertensive effects without increasing adverse event rates (26). Valsartan not only largely prevents amlodipine-induced peripheral edema but also benefits cardiovascular morbidity and mortality, with protective effects on renal function (27). Nevertheless, peripheral edema remains an undeniable adverse effect of amlodipine, and our study found it to be independent of age and gender, suggesting that combination therapy should be considered clinically to reduce its incidence. At the genetic level, specific clinical studies have found that Chinese patients carrying CYP3A5 *3/*3 or CYP3A5 *1D/*1D genotypes have a significantly increased risk of amlodipine-induced peripheral edema, while those with the CYP3A5 *1E genotype show a lower risk (28). Our study identifies peripheral edema as a noteworthy adverse reaction across all population groups, warranting further research to explore whether amlodipine-induced peripheral edema in American, European, and Asian populations is also associated with specific genotypes.

Drug-induced gingival overgrowth is a periodontal side effect of certain medications, believed to be associated with pathological growth of gingival tissue due to excessive expansion of the extracellular matrix, cell proliferation, and/or hypertrophy (29). It may be related to increased expression of IL-17A (30) and can lead to swelling, bleeding, and problems with chewing, aesthetics, and phonation, potentially resulting in tooth loss and deterioration of patients' quality of life (11). First reported in patients taking amlodipine in 1994 (31), it is now recognized as a major oral adverse reaction to the drug. Recent years have seen an increase in literature on amlodipine-induced gingival diseases, but these are mostly case reports and mechanism analyses, with few large-sample studies. Our study, based on real-world data from the FAERS database, not only confirmed gingival overgrowth but also identified rare gingival conditions including gingival swelling, gingivitis, gingival bleeding, and gingival pain. These conditions are interrelated, with one potentially triggering the simultaneous occurrence of others. Specifically, gingival hypertrophy and swelling are typically associated with inflammatory responses, which may lead to bleeding and pain. Research has emphasized that pre-existing periodontal inflammation may be a crucial factor in inducing hypertrophy (32), indicating a significant association between periodontal health status and drug-induced gingival overgrowth. Gingival inflammation is related to bacterial plaque accumulation forming microbial biofilms, and factors affecting the degree and severity of gingival swelling similarly exacerbate inflammatory responses caused by dental plaque (33). Although clinical studies report a prevalence of 3.4% for amlodipine-induced gingival overgrowth (34), our study found that associated adverse reactions are equally noteworthy, with gingival hypertrophy occurring at rates as high as 61.8%, possibly related to the upregulation of TGF-β1 and KGF gene expression (35). More significantly, in our study, gingival hypertrophy ranked highest in ROR across all five subgroup populations, suggesting that this adverse reaction requires broader clinical attention.

Shock is an acute circulatory failure state associated with infection, typically accompanied by hypotension and organ dysfunction (36). Distributive shock, a subtype, is characterized by pathological redistribution of the vascular system leading to relative hypovolemia, primarily related to vascular system dysfunction (37). It has been reported as a potential consequence of amlodipine overdose (38). In our study, distributive shock ranked among the top three adverse reactions by ROR score in all subgroups except the elderly, and may be complexly associated with other intersecting adverse reactions such as bradycardia, hypotension, left ventricular hypertrophy, and hemodynamic instability, potentially occurring simultaneously or exacerbating each other. Vasoplegia syndrome, a form of distributive shock characterized by low systemic vascular resistance including vasodilation and dysregulation of vascular smooth muscle cells (39), is commonly observed post-cardiothoracic surgery. Our study identified it as a potential adverse reaction to amlodipine, consistent with a clinical case reported by L.A.A. (40). This ADR ranked among the top five by ROR score in all subgroups except the elderly. Notably, there is currently no comprehensive analysis of the association between amlodipine and the occurrence of distributive shock and vasoplegia syndrome, underscoring the importance of early recognition, prevention, and intervention for these conditions across all populations, particularly in younger individuals.

While upper respiratory tract infections are commonly reported adverse reactions when amlodipine is combined with other antihypertensive medications (41, 42), the FDA label indicates dyspnea as a respiratory system adverse effect, consistent with our finding of dyspnoea at rest. Watt et al. (43) reported that amlodipine use may lead to increased breathlessness during exercise, but our findings suggest that attention should also be paid to dyspnea occurrence at rest. Additionally, we identified obstructive airways disorder, indicating that dyspnea may be related to airflow limitation leading to airway obstruction, though specific mechanisms require further investigation.

Given that amlodipine users are predominantly elderly, with an average age of about 68.6 years (44), we identified a noteworthy adverse reaction in the elderly subgroup: BRASH syndrome. This recently recognized clinical entity is characterized by bradycardia, renal failure, AV node blockade, shock, and hyperkalemia. Its symptoms may overlap with other conditions, making it susceptible to oversight and misdiagnosis. BRASH syndrome has significant harmful effects, with a mortality rate of 5.7%, 20% of patients requiring renal replacement therapy, and up to 33% needing temporary pacing (45). It may progress to cardiogenic shock (46). Several case reports (46, 47–49) have documented BRASH syndrome in hypertensive patients taking amlodipine, aligning with our findings. While not a common adverse reaction across all subgroups in our study, its specific symptoms such as bradycardia, shock, and AV conduction block are among the 27 common adverse reactions we identified or related to them. BRASH syndrome ranked high among adverse reactions in the elderly subgroup, indicating its potential clinical risk. Regrettably, there is currently no literature specifically discussing the association between amlodipine use and the occurrence of BRASH syndrome, particularly in the elderly population, with most reports being case studies. Our study thus serves as an important warning about the significance of monitoring this emerging entity in elderly patients. BRASH syndrome represents a vicious cycle of hyperkalemia and bradycardia, and caution should be exercised when using amlodipine in high-risk populations, such as those with a history of hyperkalemia.

Furthermore, we identified some controversial ADRs, such as effects on cardiac rhythm and left ventricular structure. The FDA label lists both bradycardia and tachycardia as adverse reactions to amlodipine. Some studies have shown increased heart rate (50) and more specifically sinus tachycardia (51) after amlodipine use, possibly due to reflex tachycardia caused by reduced peripheral vascular resistance. However, specific clinical studies have reported that combined use of angiotensin axis antagonists with amlodipine may exacerbate adverse reactions such as hypotension and bradycardia (20). A cohort study using a fixed-dose combination of bisoprolol and amlodipine also reported bradycardia as an adverse reaction (52). Although the individual effect of amlodipine was not evaluated in these studies, Ebihara et al. (53) reported a case of severe bradycardia in a patient taking a high dose of amlodipine, consistent with Mellor et al.'s (54) study suggesting that amlodipine may cause bradycardia by suppressing sympathetic nervous system activity. Combined with our results, we believe that amlodipine poses a risk of causing bradycardia and sinus bradycardia, and this risk is independent of age and gender.

Similarly, the adverse reaction of left ventricular hypertrophy is also controversial. Left ventricular hypertrophy is a change in cardiac structure and function that increases cardiac burden and may lead to serious complications such as heart failure and arrhythmias (55, 56). Amlodipine, due to its mechanism of action and its close relationship with the cardiovascular system, may lead to increased sympathetic activity, thereby affecting left ventricular structure (57). A clinical study based in Japan indicated that amlodipine can alleviate LV hypertrophy (58), and the same conclusion was drawn in hypertensive rats (59). However, an increasing body of literature has found that the effect of amlodipine in improving left ventricular hypertrophy is not as expected (60), showing its potential limitations in some hypertensive patients. A clinical study based on hypertensive populations showed that some patients using amlodipine long-term may experience morning hypertension, which is closely related to left ventricular hypertrophy (61). Takatsu et al. (62) also proposed that while amlodipine lowered blood pressure, it failed to effectively reverse indicators related to left ventricular hypertrophy, suggesting that amlodipine did not significantly improve left ventricular geometry and function. This is consistent with the study by Takeuchi et al. (63), which found that the use of amlodipine failed to effectively inhibit cardiomyocyte hypertrophy, especially in high-salt diet-induced models. Our study similarly found that amlodipine may exacerbate left ventricular hypertrophy, further emphasizing the complexity and limitations of this drug in clinical applications.

This study also identified some psychiatric ADRs, such as completed suicide, intentional overdose, and suicide attempt. We believe that suicide may be related to the decline in the patients' quality of life and increased financial burden caused by cardiovascular, oral gingival, and respiratory system diseases induced by amlodipine, which is consistent with reported cases (64). Suicide may occur through intentional overdose. Research has found that drug self-poisoning (DSP) is the most common suicide method globally, and amlodipine is ranked third among the most frequently reported drugs in this regard (65). Additionally, we identified some previously unreported ADRs, such as full blood count abnormal in the male population and personality disorder in the female population.

This study identified various ADRs associated with amlodipine, and since the FDA label has already noted most of them without in-depth discussion, such as myocardial depression, metabolic acidosis, orthostatic hypotension, and decreased diastolic blood pressure it underscores the reliability of our findings and indicates that many positive ADRs have not yet been emphasized. Drug safety is a crucial issue, and ADRs should be taken seriously. Although clinical observations and case reports make it difficult to assess whether potential ADRs associated with a drug are valid, and actual clinical observations may underestimate the prevalence of ADRs due to their low incidence rates, their potential ADRs should be thoroughly explored. As the first comprehensive investigation of the potential ADRs associated with the commonly used medication amlodipine, this study employed rigorous quality control measures, including retaining only reports where amlodipine was designated as the primary suspect and excluding reports submitted by non-healthcare professionals. Furthermore, it integrated four positive signal detection methods and validated the findings through subgroup analyses and multivariable logistics regression. Consequently, this study provides compelling evidence that amlodipine poses risks of peripheral edema, shock, and dyspnea, among others, and identified previously unreported ADRs such as abnormal full blood count and personality disorder. These findings underscore the importance of exercising caution when prescribing amlodipine to high-risk individuals with a history of hyperkalemia, cardiac structural abnormalities, or airway obstruction.

This study inevitably has some limitations. First, while we have synthesized multiple streams of evidence to identify ADRs potentially linked to amlodipine use and have offered safety recommendations for clinical application, we are unable to establish definitive causal relationships. Second, the constraints and biases inherent in the sample sources limit the generalizability of our findings across diverse ethnic populations. Third, as the data were voluntarily reported, issues such as inconsistency in quality inevitably arise. Nevertheless, we have mitigated the impact on our study by including only reports submitted by healthcare professionals. Therefore, future research should strive to explore the underlying mechanisms in greater depth and incorporate more diverse and extensive samples to comprehensively evaluate the drug's effects across different ethnic groups.

## Data Availability

The original contributions presented in the study are included in the article/Supplementary Material, further inquiries can be directed to the corresponding author.
