# Correction: Adverse events associated with amlodipine: a pharmacovigilance study using the FDA adverse event reporting system

**DOI:** 10.3389/fcvm.2025.1636315

**Published:** 2025-06-27

**Authors:** Jiazhen Jiang, Qian Zhong, Xinyu Zhou, Lisi Zhou, Jiyuan Zheng, Bingshuo Liu, Xingwei Di

**Affiliations:** ^1^ The First Clinical Medical School of Guangzhou University of Chinese Medicine, Guangzhou, Guangdong, China; ^2^ Department of Cardiovascular Medicine, First Affiliated Hospital of Jinzhou Medical University, Jinzhou, Liaoning, China; ^3^ Clinical Medical College of Acupuncture, Moxibustion and Rehabilitation, Guangzhou University of Chinese Medicine, Guangzhou, Guangdong, China; ^4^ The Fifth Clinical College, Guangzhou University of Chinese Medicine, Guangzhou, Guangdong, China; ^5^ Department of Cardiovascular Medicine, The First Affiliated Hospital of Guangzhou University of Chinese Medicine, Guangzhou, Guangdong, China

**Keywords:** amlodipine, adverse drug reactions, FDA adverse event reporting system, risk signal detection, logistic regression

A Correction on Adverse events associated with amlodipine: a pharmacovigilance study using the FDA adverse event reporting system By Jiang J, Zhong Q, Zhou X, Zhou L, Zheng J, Liu B and Di X (2025). Front. Cardiovasc. Med. 12:1504671. doi: 10.3389/fcvm.2025.1504671

The order of authors in the author list of the published paper was erroneously given as: Xingwei Di, Jiazhen Jiang, Qian Zhong, Xinyu Zhou, Lisi Zhou, Jiyuan Zheng, and Bingshuo Liu. The correct author list reads: Jiazhen Jiang, Qian Zhong, Xinyu Zhou, Lisi Zhou, Jiyuan Zheng, Bingshuo Liu and Xingwei Di. The original version of this article has been updated.

